# Supplementary material for: Altered microRNA Transcriptome in Cultured Human Liver Cells upon Infection with Ebola Virus
Source: Int J Mol Sci. 2021 Apr 6;22(7):3792. doi: 10.3390/ijms22073792 (PMC8038836; doi:10.3390/ijms22073792)
Supplement: Supplementary file 1 [file ijms-22-03792-s001.zip › Supplementary_File/D_ KEGG_Analysis_Results/16-30nt_pathway_Reston-96h-Huh7_vs_Control-96h-Huh7_down.mature_mirna_targets/hsa_EnrichmentScoreDotPlot.pdf]

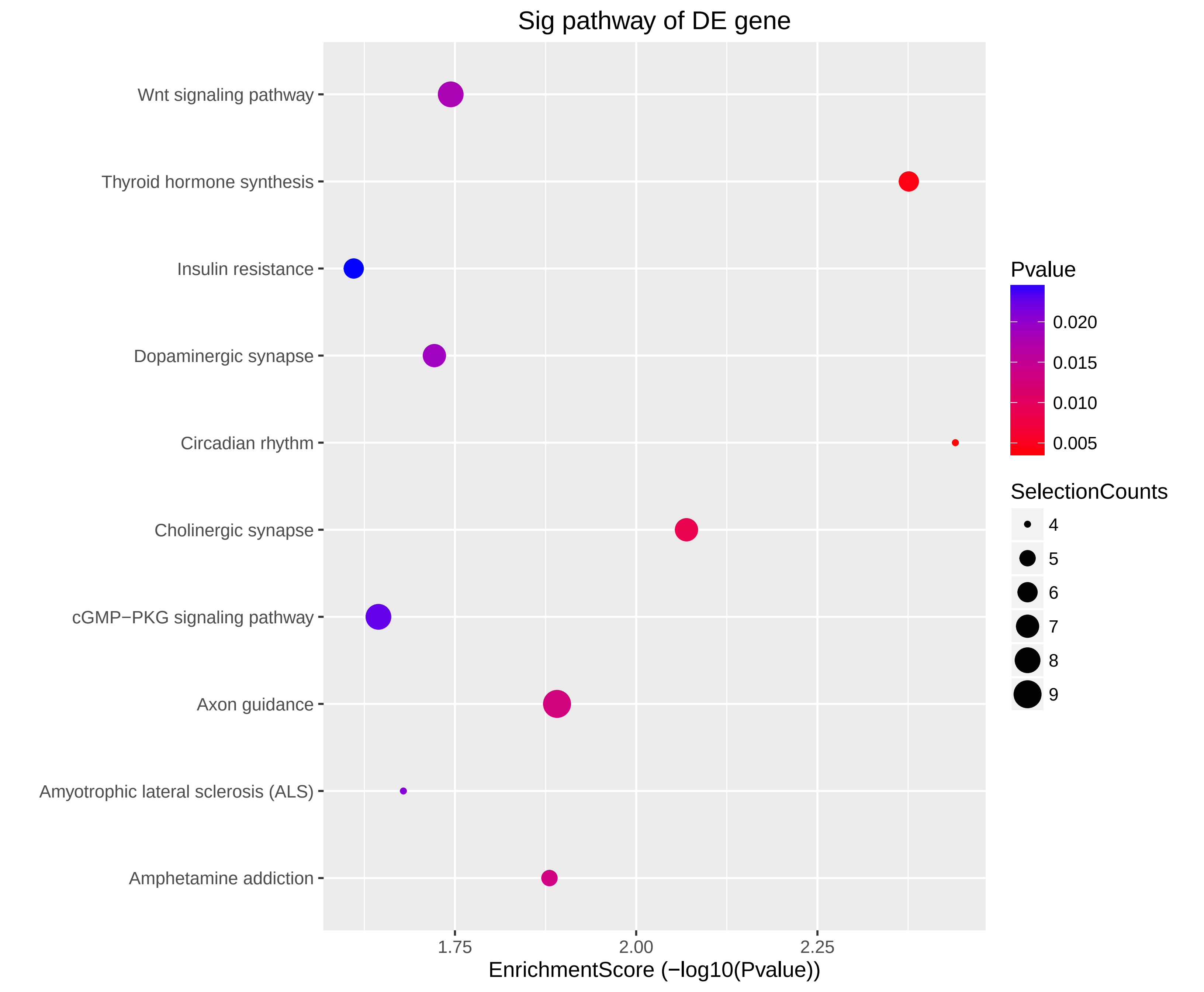

Sig pathway of DE gene
Wnt signaling pathway
Thyroid hormone synthesis
Insulin resistance
Dopaminergic synapse
Circadian rhythm
Cholinergic synapse
cGMP−PKG signaling pathway
Axon guidance
Amyotrophic lateral sclerosis (ALS)
Amphetamine addiction
1.75
2.00
2.25
EnrichmentScore (−log10(Pvalue))
Pvalue
0.020
0.015
0.010
0.005
SelectionCounts
4
5
6
7
8
9
